# Clinical, Sociodemographic, and Facility-Related Factors Influencing HER2-Targeted Therapy in Metastatic Hormone Receptor-Negative, HER2-Positive Breast Cancer

**DOI:** 10.3390/cancers17091579

**Published:** 2025-05-06

**Authors:** Ismail Ajjawi, Alejandro Rios, Wei Wei, Tristen S. Park, Maryam B. Lustberg

**Affiliations:** 1 Yale School of Medicine, Yale University, New Haven, CT 06510, USA; ismail.ajjawi@yale.edu (I.A.); alriho@gmail.com (A.R.); wei.wei@yale.edu (W.W.); 2 Mount Sinai Health System, Icahn School of Medicine, New York, NY 10018, USA; tristen.park@mountsinai.org; 3 Yale Cancer Center, Yale School of Medicine, New Haven, CT 06510, USA

**Keywords:** HER2-targeted therapy, metastatic HR−/HER2+ breast cancer, treatment disparities, survival outcomes

## Abstract

**Background/Objectives**: The use of HER2-targeted therapies has significantly improved survival outcomes in metastatic hormone receptor-negative, HER2-positive (HR−/HER2+) breast cancer. However, factors influencing their adoption remain unclear. This study examines clinical, sociodemographic, and facility-related determinants of HER2-targeted therapy utilization in metastatic HR−/HER2+ breast cancer. **Methods**: We conducted a retrospective cohort study of metastatic HR−/HER2+ breast cancer patients from the NCDB (2013–2020), categorizing them into HER2-targeted therapy recipients and non-recipients. Patients with missing key variables were excluded. Time periods were divided as pre-2015, 2016–2018, and 2019–2020 to reflect evolving treatment availability and uptake in the United States. Univariable and multivariable logistic regression identified factors associated with HER2-targeted therapy use. Cox proportional hazards regression and log-rank tests assessed overall survival. **Results**: Among 3060 metastatic HR−/HER2+ breast cancer patients, 2318 (75.8%) received HER2-targeted therapy. HER2-targeted therapy utilization increased from 64.6% in 2013 to 80.9% in 2016, marking an early period of rapid uptake. Usage remained consistently high from 2016 to 2018, followed by a slight decline and stabilization around 75% from 2019 to 2020. Factors positively associated with therapy use included diagnosis in 2016–2018 (OR 1.93, *p* < 0.001) and 2019–2020 (1.88, *p* < 0.001), private insurance (OR 1.76, *p* < 0.001), and treatment at academic facilities (OR 1.39, *p* = 0.031). Reduced likelihood of therapy use was observed in patients aged 71+ (OR 0.52, *p* < 0.001), Black race (OR 0.78, *p* = 0.018), Medicare insurance (OR 0.64, *p* < 0.001), and treatment at rural facilities (OR 0.59, *p* = 0.022). HER2-targeted therapy was associated with significantly improved survival (median 5.08 vs. 1.27 years, log-rank *p* < 0.001) and lower mortality risk (HR 0.52, *p* < 0.001). **Conclusions**: The adoption of HER2-targeted therapy has increased in recent years, yet disparities persist in access and utilization. Our findings highlight the need to address sociodemographic and facility-related barriers to ensure equitable treatment and improved survival outcomes for all patients.

## Simple Summary

Patients with metastatic HER2-positive breast cancer that is not sensitive to hormone therapy often face a rapidly progressing illness with limited options. Although the introduction of HER2-targeted therapies has led to significant improvements in survival, access to these treatments is not consistent across all patient populations. In this retrospective study of over 3000 patients using a national cancer database, we examined how factors such as age, race, insurance status, treatment facility type, and year of diagnosis influence the likelihood of receiving HER2-targeted therapy. We found that while the use of these therapies has increased over time, notable disparities remain. Younger patients, those with private insurance, and those treated at academic centers were more likely to receive targeted treatments. Importantly, patients who received HER2-targeted therapy had significantly better survival. These results underscore the importance of addressing access barriers to ensure all patients can benefit from these life-prolonging therapies.

## 1. Introduction

Metastatic hormone receptor-negative, HER2-positive (HR−/HER2+) breast cancer is a highly aggressive and difficult-to-treat subtype of metastatic breast cancer (MBC) [1]. This subtype is characterized by its rapid progression, resistance to conventional chemotherapy, and poor prognosis, posing significant challenges in clinical management [2,3,4]. The advent of HER2-targeted therapies, such as trastuzumab and pertuzumab, has revolutionized the treatment of HER2+ breast cancer, leading to significant improvements in survival outcomes for patients with metastatic disease [5,6,7]. Despite the proven efficacy of HER2-targeted therapies, the factors influencing their utilization and access in metastatic HR−/HER2+ breast cancer remain unclear.

Recent studies have highlighted the influence of clinical, sociodemographic, and facility-related factors on the use and accessibility of new cancer treatments for patients with metastatic breast cancer. For instance, Pearson et al. found that sociodemographic factors, such as younger age, white race, and higher socioeconomic status, are linked to a higher likelihood of receiving timely chemotherapy and other systemic treatments in women with metastatic breast cancer [8]. Additionally, facility-related factors play a role, as patients treated at teaching, research, and private institutions are more likely to receive timely treatment [8]. Morimoto et al. observed that clinical factors, including fewer comorbidities, more severe disease, and hormone receptor-negative status, also increase the chances of receiving chemotherapy [9]. Caswell-Jin et al. further noted that geographic location and regional income significantly affect care strategies, indicating the influence of local practice patterns and available resources [10].

Despite the expanding literature on treatment disparities in metastatic breast cancer, few studies have specifically examined the factors influencing the use of HER2-targeted therapies in patients with metastatic HR−/HER2+ breast cancer. The National Cancer Database (NCDB) offers a comprehensive dataset that includes both clinical and sociodemographic information, facilitating an in-depth analysis of treatment utilization trends, including those related to HER2-targeted therapies [11,12,13,14]. In the past decade, significant advancements in HER2-targeted treatments have transformed clinical practice in the management of metastatic HER2+ breast cancer [15,16]. These advancements include the approval of newer agents such as tucatinib in 2019 and trastuzumab deruxtecan in 2020, which expanded treatment options for patients with HR−/HER2+ metastatic breast cancer during the later years of the study period [17,18,19]. The availability and adoption of these drugs have had a profound impact on treatment patterns [17,18,19]. Analyzing NCDB data from this period presents a valuable opportunity to investigate how treatment patterns have evolved, identify factors that influence therapy selection, and assess the impact of these therapies on patient survival outcomes.

This study aims to assess the clinical, sociodemographic, and facility-related factors that influence the use of HER2-targeted therapies in patients with metastatic HR−/HER2+ breast cancer. By leveraging NCDB data, we seek to identify trends in therapy utilization, analyze disparities in access to treatment, and evaluate how these factors impact survival outcomes. The results of this study will provide critical insights for optimizing treatment strategies and improving patient care in metastatic HR−/HER2+ breast cancer.

## 2. Materials and Methods

### 2.1. Study Design and Data Source

This study used the National Cancer Database (NCDB) to conduct a retrospective analysis of patients diagnosed with metastatic HR−/HER2+ cancer between 1 January 2013, and 31 December 2020. Patients were divided into two groups: those who received HER2-targeted therapies and those who did not. This classification was based on treatment records and associated codes in the NCDB dataset. Patients were excluded if they had missing key clinical, socioeconomic, or facility-related data. Over the study period (2013–2020), HER2-targeted therapies such as trastuzumab and pertuzumab were standard first-line treatments for metastatic HER2+ breast cancer [18]. In 2019, tucatinib was approved for use in combination with trastuzumab, followed by trastuzumab deruxtecan in 2020, expanding treatment options during the later years of the study period [18].

Patients with HR+/HER2+ breast cancer were excluded to ensure a homogeneous patient population, as HR+ breast cancer requires distinct treatment approaches and has different responses to HER2-targeted therapies.

### 2.2. Patient Characteristics and Comparative Analysis

We compared demographic, clinical, and facility-related characteristics between the patients who received HER2-targeted therapies and patients who did not. Key variables included age, race, ethnicity, facility type, urbanicity, insurance status, median income, educational level by zip code, Charlson Comorbidity Index score, metastatic site, treatment at multiple facilities, other treatments, and year of diagnosis.

### 2.3. Data Analysis

Univariate comparisons and multivariate logistic regression were performed to identify factors associated with use of HER2-targeted therapies. Independent variables included age, race, ethnicity, facility type, urbanicity, insurance status, median income, educational level by zip code, Charlson Comorbidity Index score, metastatic site, treatment at multiple facilities, other treatments, and year of diagnosis. Chi-squared tests were used for comparing categorical variables, and *t*-tests were applied to continuous variables.

Kaplan–Meier survival curves were generated to compare overall survival between patients who received HER2-targeted therapies and those who did not, with the log-rank test used to test for significance. Cox regression was conducted to assess the impact of use of HER2-targeted therapies on overall survival while controlling for clinical, sociodemographic, and facility-related characteristics.

Statistical analyses were conducted using RStudio (Version 2023.12.0 + 369). A *p*-value of <0.05 was considered statistically significant.

## 3. Results

### 3.1. Trends in Use of HER-2 Targeted Therapy for Metastatic HR−/HER2+ Breast Cancer

**Figure 1:**
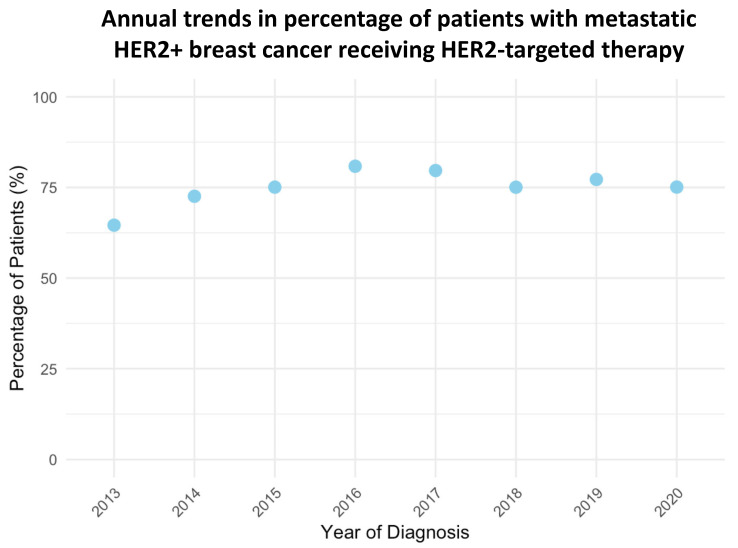
Annual trends in the use of HER2-targeted therapies among metastatic HR−/HER2+ breast cancer patients from 2013 to 2020 (*p* < 0.001 by fitting the data to a linear trend).

The use of HER-2 targeted therapy for metastatic HR−/HER2+ breast cancer increased consistently from 64.6% in 2013 to 80.9% in 2016 (Figure 1, *p* < 0.001). Although there was a slight decline in 2017 (79.7%), the percentage remained high through 2020, stabilizing at 75.1%. This trend reflects the growing adoption of HER-2 targeted therapy over the study period.

### 3.2. Patient and Treatment Facility Characteristics

A total of 3060 patients with metastatic HER2-positive breast cancer were identified from the NCDB between 2013 and 2020. Of these, 2318 (75.8%) received HER2-targeted therapy, while 742 (24.2%) did not (Table 1).

Patients who received HER2-targeted therapy were generally younger, with a higher percentage in the 40–54 (34.0% vs. 19.6%, *p* < 0.001) and 55–70 (48.8% vs. 44.5%, *p* < 0.001) age groups, and fewer in the 71+ group (17.1% vs. 35.9%, *p* < 0.001). Use of HER2-targeted therapy was more common among patients diagnosed between 2016 and 2018 (41.4%) and 2019 and 2020 (32.3%) compared to those diagnosed between 2013 and 2015 (26.2%, *p* < 0.001).

Racial differences were observed, with a higher proportion of HER2-targeted therapy patients being White (77.0% vs. 73.1%, *p* = 0.041) and fewer being Black (16.6% vs. 20.2%, *p* = 0.041). Hispanic ethnicity was slightly more common among HER2-targeted therapy patients (6.4% vs. 6.2%, *p* = 0.004). Insurance status varied significantly, with HER2-targeted therapy patients more likely to have private insurance (48.7% vs. 28.3%, *p* < 0.001) and Medicaid (14.0% vs. 12.2%, *p* < 0.001), while those not receiving HER2-targeted therapy were more often covered by Medicare (51.5% vs. 31.0%, *p* < 0.001) or uninsured (5.0% vs. 4.3%, *p* < 0.001). Also, patients receiving HER2-targeted therapy had higher median incomes, with a greater proportion earning ≥ USD 46,000 (38.3% vs. 33.8%, *p* < 0.001) and fewer earning < USD 30,000 (9.7% vs. 18.1%, *p* < 0.001).

Regarding facility-related factors, HER2-targeted therapy patients were more likely to be treated at academic (36.2% vs. 26.3%, *p* < 0.001) and comprehensive cancer centers (36.0% vs. 40.3%, *p* < 0.001), and less likely to be treated at community facilities (7.2% vs. 12.1%, *p* < 0.001). A higher percentage of HER2-targeted therapy patients were treated at more than one Commission on Cancer (CoC) facility (20.6% vs. 12.2%, *p* < 0.001).

Regarding clinical factors, patients receiving HER2-targeted therapy had lower Charlson Comorbidity Index scores, with a greater proportion having a score of 0 (84.3% vs. 74.9%, *p* < 0.001) and fewer having a score of ≥3 (1.5% vs. 5.2%, *p* < 0.001). Metastatic patterns varied, with brain metastases being less common in HER2-targeted therapy patients (5.1% vs. 9.5%, *p* < 0.001), while no significant differences were observed in bone, liver, or lung metastases. Additionally, HER2-targeted therapy patients were far more likely to receive chemotherapy (86.6% vs. 37.8%, *p* < 0.001) and surgery at the primary tumor site (25.4% vs. 13.1%, *p* < 0.001).

### 3.3. Factors Associated with HER2-Targeted Therapy Use in Metastatic HR−/HER2+ Breast Cancer

A multivariate logistic regression analysis identified key factors influencing the likelihood of receiving HER-2 targeted therapy among patients with HER2+ breast cancer (Table 2). Age was a significant factor, with patients aged 71 and older being significantly less likely to receive HER-2 therapy compared to those aged 40–54 (OR = 0.41, 95% CI: 0.30–0.57, *p* < 0.001). Patients aged 55–70 were also less likely to receive HER-2 therapy, though the association was weaker (OR = 0.76, 95% CI: 0.60–0.96, *p* = 0.025). The year of diagnosis was another significant factor, with patients diagnosed between 2016 and 2018 (OR = 1.93, 95% CI: 1.40–2.65, *p* < 0.001) and those diagnosed between 2019 and 2020 (OR = 1.88, 95% CI: 1.35–2.62, *p* < 0.001) having higher odds of receiving HER-2 therapy compared to those diagnosed between 2013 and 2015.

Regarding socioeconomic factors, racial disparities were observed, with Black patients being less likely to receive HER-2 therapy compared to White patients (OR = 0.81, 95% CI: 0.67–0.95, *p* = 0.043). Insurance status was strongly associated with receiving HER-2 therapy, with patients having private insurance being more likely to receive treatment compared to uninsured individuals (OR = 1.79, 95% CI: 1.17–2.73, *p* = 0.007). Income also played a role, with higher median income associated with increased odds of receiving HER-2 therapy. Patients in the USD 30,000–USD 34,999 income range (OR = 1.75, 95% CI: 1.19–2.56, *p* = 0.004), USD 35,000–USD 45,999 income range (OR = 1.65, 95% CI: 1.11–2.47, *p* = 0.004), and ≥USD 46,000 income range (OR = 1.78, 95% CI: 1.24–2.76, *p* = 0.010) were more likely to receive HER-2 therapy compared to those with an income below USD 30,000.

Regarding facility-related factors, patients treated at academic centers were significantly more likely to receive HER-2 therapy compared to those treated at community centers (OR = 2.57, 95% CI: 1.77–3.73, *p* < 0.001). Patients treated at comprehensive and network facilities also had higher odds of receiving HER-2 therapy compared to those treated at community centers (OR = 1.47, 95% CI: 1.03–2.10, *p* = 0.035; OR = 1.73, 95% CI: 1.17–2.56, *p* = 0.005, respectively).

Regarding clinical factors, metastatic sites were significant predictors of HER-2 therapy use. Patients with brain metastasis were significantly less likely to receive HER-2 therapy compared to those without brain metastasis (OR = 0.43, 95% CI: 0.29–0.64, *p* < 0.001). Additionally, patients receiving chemotherapy were more likely to receive HER-2 therapy (OR = 9.53, 95% CI: 7.65–11.86, *p* < 0.001), while surgery at the primary site was not significantly associated (OR = 1.28, 95% CI: 0.97–1.68, *p* = 0.083). The Charlson Comorbidity Index score was also an important factor, with patients having a score of ≥3 being less likely to receive HER-2 therapy compared to those with a score of 0 (OR = 0.40, 95% CI: 0.22–0.73, *p* = 0.002).

### 3.4. Impact of HER2-Targeted Therapy Use on Survival

**Figure 2:**
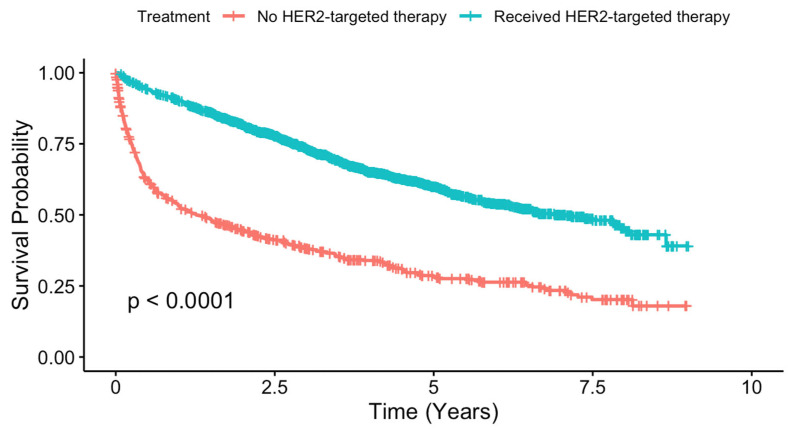
Kaplan–Meier survival curves comparing overall survival in metastatic HR−/HER2+ breast cancer patients receiving HER2-targeted therapies versus those not receiving HER2-targeted therapies.

The impact of HER-2 targeted therapy on survival was significant, with Kaplan–Meier analysis showing improved survival for patients receiving HER-2 targeted therapy compared to those who did not (Figure 2, *p* < 0.001). Non-HER-2 therapy patients had a median survival time of 1.27 years (95% CI: 0.97–1.82), while HER-2 therapy patients had a substantially longer median survival time of 5.08 years (95% CI: 4.25–6.03).

Cox regression, adjusted for clinical, sociodemographic, and facility-related factors, confirmed a significant survival benefit for patients receiving HER-2 targeted therapy (HR: 0.52, 95% CI: 0.45–0.59, *p* < 0.001) (Table 3). Additional factors influencing survival included age, with patients aged 55–70 (HR: 1.42, 95% CI: 1.21–1.67, *p* < 0.001) and 71+ (HR: 1.62, 95% CI: 1.30–2.02, *p* < 0.001) having higher mortality risks compared to those aged 40–54. Year of diagnosis also played a role, with patients diagnosed in 2014–2017 (HR: 0.75, 95% CI: 0.62–0.92, *p* = 0.005) and 2018–2020 (HR: 0.84, 95% CI: 0.72–0.96, *p* = 0.021) showing improved survival. Race was another factor, with Black patients (HR: 1.22, 95% CI: 1.04–1.43, *p* = 0.014) and those from other racial groups (HR: 0.59, 95% CI: 0.39–0.89, *p* = 0.012) having significantly different survival outcomes. The presence of bone (HR: 1.18, 95% CI: 1.00–1.39, *p* = 0.042), brain (HR: 1.91, 95% CI: 1.48–2.46, *p* < 0.001), and liver (HR: 1.49, 95% CI: 1.27–1.76, *p* < 0.001) metastases was associated with increased mortality, while chemotherapy (HR: 0.38, 95% CI: 0.33–0.44, *p* < 0.001) and surgery at the primary site (HR: 0.57, 95% CI: 0.47–0.67, *p* < 0.001) were linked to improved survival. Other significant factors included insurance status, with patients having private insurance showing improved survival (HR: 0.67, 95% CI: 0.50–0.89, *p* = 0.005), and treatment at more than one Commission on Cancer (CoC)-accredited facility, which also showed a survival advantage (HR: 0.84, 95% CI: 0.72–1.00, *p* = 0.048).

## 4. Discussion

The results of this study emphasize the evolving patterns of HER2-targeted therapy use in patients with metastatic hormone receptor-negative, HER2-positive (HR−/HER2+) breast cancer. From 2013 to 2020, we observed a marked increase in the use of HER2-targeted therapies, reflecting advancements in treatment options and clinical practice. The recommendations from the ABC conferences since 2011 have played a pivotal role in shaping international guidelines and treatment strategies, potentially contributing to this growing usage of HER2-targeted therapies [20]. Despite the growing adoption of these therapies, disparities persist in their utilization, with significant differences in access based on sociodemographic and facility-related factors. These findings highlight critical issues related to equity in cancer care and provide a basis for future strategies aimed at ensuring more inclusive access to advanced therapies.

Our analysis confirms previous observations that clinical factors such as age, comorbidity, and disease severity influence the likelihood of receiving HER2-targeted therapies [8,9]. Younger patients and those with fewer comorbidities were more likely to receive HER2-targeted therapy, a pattern that is consistent with the broader trend of younger, healthier individuals benefiting more frequently from aggressive cancer treatments [21,22]. This may reflect both clinical decision-making practices and the consideration of the patient’s ability to tolerate complex therapies, which is a crucial factor in treatment selection for metastatic breast cancer.

Racial and ethnic disparities in treatment access were also evident in our study. White patients were more likely to receive HER2-targeted therapy compared to Black patients, which mirrors broader trends of racial disparities in cancer care that have been widely documented [8,10]. These findings are concerning, as they highlight potential barriers that minority populations face in accessing cutting-edge therapies, which may be linked to systemic inequities in healthcare delivery and patient–provider interactions.

Socioeconomic factors such as insurance status and income were significant predictors of HER2-targeted therapy utilization. Patients with private insurance were more likely to receive HER2-targeted therapy compared to the uninsured, which is in line with previous studies that suggest insurance coverage plays a pivotal role in access to advanced cancer treatments [23,24]. In the United States, trastuzumab is generally covered by most insurance plans, including private insurance, Medicare, and Medicaid. However, coverage can vary significantly depending on the specific insurance provider, plan type, and geographic location [25]. Private insurance plans may require prior authorization or completion of step therapy protocols, which can delay treatment access. Medicare provides coverage for trastuzumab, though beneficiaries may face substantial out-of-pocket costs, including coinsurance and deductibles. Medicaid coverage is state-dependent, leading to disparities in access across different regions. Additionally, a significant portion of the population remains uninsured, and these individuals may face considerable financial barriers to accessing trastuzumab. While patient assistance programs can alleviate some of these costs, their availability is not uniform, and many individuals remain unable to afford the treatment. Similarly, patients from higher-income backgrounds had greater access to these therapies, indicating that financial resources may continue to be a substantial barrier to care for lower-income populations. Trastuzumab, despite the availability of biosimilars, remains costly, and access is highly dependent on socioeconomic status and insurance coverage [26,27]. This disparity in access underscores the importance of addressing financial and insurance-related barriers to ensure that all patients, regardless of their socioeconomic status, can benefit from the latest therapeutic advancements.

Geographic location also emerged as a key determinant of treatment access. Patients treated at academic centers, and those residing in urban areas were more likely to receive HER2-targeted therapy, reflecting the concentration of advanced treatments and specialized care in certain healthcare settings [28,29,30]. This finding reinforces the notion that healthcare infrastructure plays a critical role in determining treatment availability. Rural patients, who often face challenges in accessing specialized care, were less likely to receive HER2-targeted therapies, which points to a need for targeted interventions aimed at improving access to such therapies in rural and underserved regions.

The survival outcomes associated with HER2-targeted therapy in our study were promising. Kaplan–Meier analysis demonstrated that patients who received HER2-targeted therapy had significantly improved survival compared to those who did not. This improvement aligns with clinical trial results, such as those from the CLEOPATRA trial, which showed the survival benefits of combining HER2-targeted therapies with chemotherapy for metastatic HER2-positive breast cancer [5]. These survival benefits underscore the importance of ensuring equitable access to HER2-targeted therapies, as they have the potential to significantly improve patient outcomes in this aggressive cancer subtype.

Despite the robust findings of this study, there are several limitations. One key limitation is that the NCDB provides information on whether a tumor is classified as HER2-positive or HER2-negative, but it does not include detailed data on HER2 expression levels (e.g., HER2+++). Since HER2 expression levels are important for determining treatment decisions, including the potential higher likelihood of recommendation of HER2-targeted therapies for patients with higher HER2 expression, the lack of this granular information may have impacted survival outcomes. This limitation means that we were unable to account for the influence of HER2 expression levels on treatment decisions and outcomes in our regression models. Additionally, while the NCDB includes a wide range of clinical and sociodemographic data, it does not capture certain factors, such as treatment response or patient preferences, that could influence treatment decisions. Furthermore, the NCDB’s focus on Commission on Cancer-accredited facilities may limit the generalizability of our findings to non-academic or rural settings, where treatment patterns may differ. Additionally, the NCDB does not distinguish between different HER2-targeted agents, such as trastuzumab alone versus trastuzumab combined with pertuzumab, which limits our ability to analyze the specific treatment regimens and their associations with survival outcomes. Moreover, a notable limitation of our study is the inability to determine the exact reasons why approximately 25% of patients with metastatic HR−/HER2+ breast cancer did not receive HER2-targeted therapy, despite its established survival benefit.

## 5. Conclusions

In conclusion, while the use of HER2-targeted therapies in metastatic HR−/HER2+ breast cancer has significantly increased over the past decade, our study highlights critical disparities in access to this promising treatment. Clinical, sociodemographic, and facility-related factors all play substantial roles in determining which patients benefit from HER2-targeted therapies. These inequities suggest a need for more targeted policies and interventions aimed at reducing barriers to treatment, particularly for underserved populations [31]. Future research should focus on identifying the underlying causes of these disparities and exploring strategies to improve access, such as expanding insurance coverage, enhancing healthcare delivery in rural areas, and ensuring that patients with higher comorbidities are not excluded from potentially life-saving therapies. By addressing these gaps, we can ensure that the benefits of HER2-targeted therapies reach all patients, ultimately improving survival outcomes and promoting greater health equity in the management of metastatic HR−/HER2+ breast cancer.

## Figures and Tables

**Table 1:** Demographic, clinical, socioeconomic, and treatment facility characteristics of patients with metastatic HER2+ breast cancer and correlation with HER2-targeted therapies use.

	No HER2-Targeted Therapies (N = 742)	Received HER2-Targeted Therapies (N = 2318)	*p*-Value
Age group			<0.001
40–54	146 (19.6%)	789 (34.0%)	
55–70	332 (44.5%)	1132 (48.8%)	
71+	268 (35.9%)	397 (17.1%)	
Year of Diagnosis			<0.001
2013–2015	247 (33.1%)	608 (26.2%)	
2016–2018	264 (35.4%)	960 (41.4%)	
2019–2020	235 (31.5%)	750(32.3%)	
Race (%)			0.041
White	545 (73.1%)	1784 (77.0%)	
Black	151 (20.2%)	385 (16.6%)	
Asian	12 (1.6%)	42 (1.8%)	
South Asian	25 (3.4%)	82 (3.5%)	
Other	4 (0.5%)	14 (0.6%)	
Unknown	9 (1.2%)	11 (0.5%)	
Hispanic/Spanish Origin (%)			0.004
Yes	46 (6.2%)	148 (6.4%)	
No	677 (90.8%)	2140 (92.3%)	
Unknown	23 (3.1%)	30 (1.3%)	
Facility Type (%)			<0.001
Community	90 (12.1%)	167 (7.2%)	
Comprehensive	301 (40.3%)	835 (36.0%)	
Academic	196 (26.3%)	840 (36.2%)	
Network	159 (21.3%)	476 (20.5%)	
Urbanicity (%)			
Metropolitan	614 (82.3%)	1909 (82.4%)	0.541
Rural	27 (3.6%)	103 (4.4%)	
Urban	105 (14.1%)	306 (13.2%)	
Insurance Status (%)			<0.001
Not Insured	37 (5.0%)	100 (4.3%)	
Private Insurance	211 (28.3%)	1130 (48.7%)	
Medicaid	91 (12.2%)	325 (14.0%)	
Medicare	384 (51.5%)	719 (31.0%)	
Other Government	7 (0.9%)	27 (1.2%)	
Unknown	16 (2.1%)	17 (0.7%)	
Treatment at >1 CoC facility (%)			<0.001
Yes	91 (12.2%)	477 (20.6%)	
No	655 (87.8%)	1841 (79.4%)	
Median Income (%)			<0.001
<USD 30,000	135 (18.1%)	226 (9.7%)	
USD 30,000–USD 34,999	99 (13.3%)	306 (13.2%)	
USD 35,000–USD 45,999	143 (19.2%)	529 (22.8%)	
≥USD 46,000	252 (33.8%)	887 (38.3%)	
Unknown	117 (15.7%)	370 (15.9%)	
Charlson Comorbidity Index Score (%)			<0.001
0	559 (74.9%)	1954 (84.3%)	
1	112 (15.0%)	264 (11.4%)	
2	36 (4.8%)	65 (2.8%)	
≥3	39 (5.2%)	35 (1.5%)	
Metastatic Site (%)			
Bone	301 (40.3%)	998 (43.0%)	0.208
Brain	71 (9.5%)	118 (5.1%)	<0.001
Liver	188 (25.2%)	652 (28.1%)	0.130
Lung	190 (25.4%)	526 (22.7%)	0.131
Other	100 (13.4%)	190 (8.2%)	<0.001
Other Treatments (%)			
Chemotherapy	282 (37.8%)	2008 (86.6%)	<0.001
Surgery at primary site	98 (13.1%)	589 (25.4%)	<0.001

**Table 2:** Multivariate logistic regression analysis of factors associated with HER2-targeted therapies use in metastatic HER2+ breast cancer patients.

	OR (95% CI)	*p*-Value
Age group		
40–54	REF	
55–70	0.76 (0.60–0.96)	0.025
71+	0.41 (0.30–0.57)	<0.001
Year of Diagnosis		
2013–2015	REF	
2016–2018	1.93 (1.40–2.65)	<0.001
2019–2020	1.88 (1.35–2.62)	<0.001
Race		
White	REF	
Black	0.81 (0.67–0.95)	0.043
Asian	0.92 (0.44–1.94)	0.831
South Asian	0.85 (0.22–3.27)	0.817
Other	0.91 (0.53–1.59)	0.751
Hispanic/Spanish Origin		
No	REF	
yes	0.77 (0.51–1.17)	0.222
Facility Type		
Community	REF	
Comprehensive	1.47 (1.03–2.10)	0.035
Academic	2.57 (1.77–3.73)	<0.001
Network	1.73 (1.17–2.56)	0.005
Urbanicity (%)		
Metropolitan	REF	
Rural	1.30 (0.77–2.19)	0.326
Urban	1.13 (0.83–1.53)	0.455
Insurance Status (%)		
Not Insured	REF	
Private Insurance	1.79 (1.17–2.73)	0.007
Medicaid	1.26 (0.74–2.15)	0.393
Medicare	0.96 (0.57–1.62)	0.884
Other Government	1.09 (0.36–3.35)	0.875
Median Income (%)		
<USD 30,000	REF	
USD 30,000–USD 34,999	1.75 (1.19–2.56)	0.004
USD 35,000–USD 45,999	1.65 (1.11–2.47)	0.004
≥USD 46000	1.78 (1.24–2.76)	0.010
Charlson Comorbidity Index Score (%)		
0	REF	
1	0.84 (0.63–1.13)	0.247
2	1.07 (0.65–1.75)	0.799
≥3	0.40 (0.22–0.73)	0.002
Treatment at >1 CoC facility (%)		
No	REF	
Yes	1.46 (1.10–1.94)	0.008
Metastatic Site (%)		
Bone	0.99 (0.78–1.28)	0.976
Brain	0.43 (0.29–0.64)	<0.001
Liver	0.98 (0.76–1.26)	0.876
Lung	1.00 (0.77–1.29)	0.992
Other	0.46 (0.27–0.89)	0.008
Other Treatments (%)		
Chemotherapy	9.53 (7.65–11.86)	<0.001
Surgery at primary site	1.28 (0.97–1.68)	0.083

**Table 3:** Cox regression to identify factors influencing overall survival (OS) in patients with metastatic HR−/HER2+ breast cancer.

	OR (95% CI)	*p*-Value
Received HER2-targeted therapy		
No	REF	
Yes	0.52 (0.45–0.59)	<0.001
Age group		
40–54	REF	
55–70	1.42 (1.21–1.67)	<0.001
71+	1.62 (1.30–2.02)	<0.001
Year of Diagnosis		
2013–2015	REF	
2016–2018	0.75 (0.62–0.92)	0.005
2019–2020	0.84 (0.72- 0.96)	0.021
Race		
White	REF	
Black	1.22 (1.04–1.43)	0.014
Asian	1.31 (0.87–1.98)	0.201
South Asian	1.81 (0.74–4.42)	0.193
Other	0.59 (0.39–0.89)	0.012
Hispanic/Spanish Origin		
No	REF	
yes	0.71 (0.43–1.02)	0.065
Facility Type		
Community	REF	
Comprehensive	0.98 (0.79–1.22)	0.874
Academic	0.84 (0.67–1.05)	0.132
Network	0.97 (0.77–1.23)	0.816
Urbanicity (%)		
Metropolitan	REF	
Rural	1.03 (0.76–1.41)	0.841
Urban	1.01 (0.85–1.22)	0.879
Insurance Status (%)		
Not Insured	REF	
Private Insurance	0.67 (0.50–0.89)	0.005
Medicaid	0.85 (0.62–1.16)	0.312
Medicare	0.90 (0.67–1.21)	0.483
Other Government	0.63 (0.31–1.29)	0.215
Median Income (%)		
<USD 30,000	REF	
USD 30,000–USD 34,999	1.14 (0.90–1.44)	0.257
USD 35,000–45,999	1.09 (0.87–1.39)	0.429
≥USD 46,000	0.82 (0.66–1.02)	0.078
Charlson Comorbidity Index Score (%)		
0	REF	
1	1.05 (0.88–1.25)	0.604
2	1.34 (0.98–1.82)	0.073
≥3	1.37 (0.97–1.94)	0.072
Treatment at >1 CoC facility (%)		
No	REF	
Yes	0.84 (0.72–1.00)	0.048
Metastatic Site (%)		
Bone	1.18 (1.00–1.39)	0.042
Brain	1.91 (1.48–2.46)	<0.001
Liver	1.49 (1.27–1.76)	<0.001
Lung	1.07 (0.90–1.26)	0.452
Other	1.50 (1.21–1.85)	<0.001
Other Treatments (%)		
Chemotherapy	0.38 (0.33–0.44)	<0.001
Surgery at primary site	0.57 (0.47–0.67)	<0.001

## Data Availability

This study utilized the publicly available, de-identified National Cancer Database (NCDB), which can be accessed through the American College of Surgeons and the American Cancer Society upon application and approval.
